# Potential of Extracted *Locusta Migratoria* Protein Fractions as Value-Added Ingredients

**DOI:** 10.3390/insects9010020

**Published:** 2018-02-09

**Authors:** Claudia Clarkson, Miranda Mirosa, John Birch

**Affiliations:** Food Science Department, University of Otago, PO Box 56, Dunedin 9054, New Zealand; cclarkson.nz@gmail.com (C.C.); miranda.mirosa@otago.ac.nz (M.M.)

**Keywords:** entomophagy, *Locusta migratoria*, insect proteins, fatty acid, functionality, colour, proximate composition, food

## Abstract

Although locusts can be sustainably produced and are nutrient rich, the thought of eating them can be hard to swallow for many consumers. This paper aims to investigate the nutritional composition of *Locusta migratoria*, including the properties of extracted locust protein, contributing to limited literature and product development opportunities for industry. Locusts sourced from Dunedin, New Zealand, contained a high amount of protein (50.79% dry weight) and fat (34.93%), which contained high amounts of omega-3 (15.64%), creating a desirably low omega-3/omega-6 ratio of 0.57. Three protein fractions including; insoluble locust fraction, soluble locust fraction, and a supernatant fraction were recovered following alkali isoelectric precipitation methodology. Initially, proteins were solubilised at pH 10 then precipitated out at the isoelectric point (pH 4). All fractions had significantly higher protein contents compared with the whole locust. The insoluble protein fraction represented 37.76% of the dry weight of protein recovered and was much lighter in colour and greener compared to other fractions. It also had the highest water and oil holding capacity of 5.17 mL/g and 7.31 mL/g, possibly due to larger particle size. The high supernatant yield (56.60%) and low soluble protein yield (9.83%) was unexpected and could be a result of experimental pH conditions chosen.

## 1. Introduction

In recent years, there has been a growing interest into the benefits of consuming insects, also known as entomophagy. Instead of viewing insects, such as locusts, as pests that should be kept away from the kitchen, certain consumers and food companies are purposely and creatively incorporating them into food products and meals. Migratory locusts (*Locusta migratoria*, Linnaeus, 1758 (Orthoptera: Acrididae)) are prevalent in African and Arabian diets, but are also widespread across the world [1]. Popular preparations include fried, roasted, or boiled. However, there are over 2000 edible insect species that can be eaten at egg, larval, pupal, or adult stages, either whole or ground up into meals or snack products [2]. Locusts are historically associated as pests that destroy crops, which has influenced research and consumer opinions. In recent years however, through growing interest concerning entomophagy, locusts have received attention for their promising sensory properties, nutritionally rich composition, and sustainable production prospects as a food ingredient [1,2].

Rearing locusts and other edible insects requires significantly less feed, land, and water compared to livestock production, which is known to have a significant impact on the environment where greenhouse gas emissions are minimal with only cockroaches, termites and scarab beetles that emit methane gas [2,3]. Locusts have short life cycles in which adulthood can be reached at four to eight weeks depending on rearing conditions. They can also increase their numbers 10 to 16-fold between each generation, illustrating the rapid and efficient domestic rearing prospects [1]. Land required for rearing is minimal as they can be farmed vertically. The cold-blooded insects obtain moisture from their food decreasing water requirements, and along with the high feed conversion efficiency, insects resource requirements are lower compared to other traditional livestock such as beef [2].

Moreover, locusts are nutritionally rich in protein, fiber, and essential fatty acids. Compositional data concerning locusts is scarce and exact values are highly variable depending on species, habitat, diet, metamorphic stage, and processing method. Orthopteran species contain high amounts of fat averaging around 13% (dry weight basis), with promising amounts of essential polyunsaturated fatty acids (PUFA); omega-3 (α-linolenic acid) and omega-6 (linoleic acid) [4]. Moreover, insects are particularly noted for their high protein content. Orthopteran species can range from 15% to 81% protein [5], with averages around 50% to 65% for *Locusta migratoria* [6,7,8]. Orthopteran species also contain satisfactory ratios of essential amino acids recommended for human consumption, making them a sustainable protein option for consumers looking for alternatives to traditional meat sources such as beef [5]. Yet, some studies have recognised the potential overestimation of insect protein contents when the nitrogen to protein conversion for meat (6.25) is used [9,10,11]. The high amount of chitin, a non-protein nitrogen (NPN) polysaccharide found in the exoskeleton of insects, can cause an overestimation of digestible protein documented in literature. To more accurately determine insect protein content, Jonas-Levi and Martinez [10] recommended subtracting NPN from the total nitrogen content, or lowering the conversion factor. Nonetheless, the high protein and fat content of insects and locusts is comparable to traditional meat sources, indicating potential as a meat or protein alternative for the growing population [2,12].

Furthermore, extracting the protein from insects has been suggested as a worthwhile practice [2]. Not only does it increase the protein content (per 100 g) and digestibility for certain fractions [13], it can overcome initial consumer acceptance barriers. Many studies have found consumers are more willing to eat insects when they cannot be seen [14], illustrating opportunities as an value-added ingredient as opposed to a whole insect. Protein extraction is usually carried out via aqueous or alkali isoelectric precipitation, in which different pH, temperature, and physical separation (centrifugation) methods are used to separate different fractions [15]. These fractions include a layer comprising of water insoluble proteins and other constituents, and a layer of protein water soluble proteins. Each fraction is different in terms of yield, chemical composition, digestibility, colour, and functionality. Consequently, insoluble and soluble proteins have different potential applications as an ingredient. To the authors knowledge, only a small number of studies have explored any of these parameters, which include analysis of house crickets *(Acheta domesticus),* black solider flies *(Hermeia illucens)*, yellow mealworms *(Tenebrio molitor*), Mexican fruit fly larvae *(Anastepha ludens)* and migratory locusts (*Locusta migratoria*) [11,15,16,17,18,19].

Insects in New Zealand, including locusts, are classified as novel food by Food Standards Australia New Zealand (FSANZ), with the exception of three species, due to lack of safety concerns. Studies have found microbial and fungal loads of fresh insects are reasonably high [20,21,22]. However, processing steps such as blanching, heat treatments, and sterilization are sufficient methods to reduce safety risks in various insect species [20,22,23].

Presently, there are only a few commercial insect products sold on the Western market, which include ground insect flour (often cricket), sold on its own or incorporated into bars, chips, protein powders, cookies and even pasta. Locusts are less popular and very few products have utilised extracted insect protein as an ingredient. Even with the promising potential of locusts as an alternative food or protein source, consumers must first be accepting of the idea. Entomophagy is practiced by over two billion people in mostly African, Asian, and Latin American societies [2]. Nevertheless, in areas where they are not traditionally consumed, such as Western culture, eating insects is met with great resistance, surfacing feelings of disgust and fear [24]. Enculturation of viewing animal products as a protein and fat source, and viewing insects as a pest that people try to avoid, has caused avoidance of novel insect products, also known as food neophobia. In order to successfully introduce locust products, greater understanding of the locust protein properties could help the limited research and industrial product development, in the hope to improve adoption in the future. Therefore, the present study aimed to investigate the proximate and fatty acid composition of the adult migratory locusts (*Locusta migratoria*) and the properties, yield, and composition of extracted protein fractions via alkali isoelectric precipitation methodology.

## 2. Materials and Methods

### 2.1. Preparation of Locust Meal

Whole adult *Locusta migratoria* (4 weeks) were donated by Otago Locusts, Dunedin, NZ. The locusts were reared in large plastic containers at a constant ambient temperature and light source. The feed consisted of oat grass and ground oats. Live locusts were hand harvested into small containers and placed into a −18 °C freezer overnight. Whole samples were freeze-dried (Freezezone 12 Plus, Labconco, Kansas, MO, USA) and ground to produce locust meal (LM). Defatted locust meal (DLM) was prepared by dispersing LM in hexane (1:5, *w*/*v*) and stirred for three hours using a magnetic stirrer (S-12BB, Jeio Tech Ltd., Seoul, South Korea), with renewal of hexane every hour. The mixture was filtered thrice, and dried in a fume hood to remove any remaining organic solvent. LM and DLM were stored in a desiccator at room temperature until analysis. General chemical materials used in the study were analytical grade and acquired from Thermo Fisher, Sciresby, VIC, Australia.

### 2.2. Proximate Analysis

Chemical analysis of the LM were determined based on the Association of Analytical Chemists official methods [25]. Moisture was calculated on a weight basis after freeze-drying whole locust for seven days. Crude fat was extracted from LM (2 g) for 1.5 h in Soxhlet apparatus, using hexane. Fat content was determined gravimetrically after the solvent was evaporated off using a Rotary evaporator (Rotovapor-R, Buchi, Flawil, Switzerland) and a room temperature water bath (Watson Victor Ltd., Buchi 461 Water Bath, Auckland, New Zealand). Crude protein was determined using the Kjeldahl method (Tecator Digestion System 1007 Digester; Kjeltec System 1002 Distilling Unit, Mulgrave, VIC, Australia), with a 6.25 N conversion factor. Ash content was found gravimetrically after drying the sample in a muffle furnace (McGregor, Interlab, Wellington, New Zealand) at 600 °C for eight hours. Carbohydrates were determined by difference. Analysis was carried out in triplicate.

### 2.3. Fatty Acid Composition

FA composition was determined from fatty acid methyl esters (FAME), prepared following a modified method of Van Wijngaarden [26]. Locust oil (20 mg) and internal standard (C17:0; 10 mg/mL) was mixed with methanolic KOH and heated for 20 min at 80 °C in a heating block (Grant BT3, Grant Instruments Ltd., Cambridge, UK). After addition of diethyl ether (2 mL) and water (5 mL), the bottom layer was acidified using HCl (36.5–38%) and a further aliquot of diethyl ether (2 mL) was added. The diethyl ether upper phase was added into boron triflouride-methanol solution (2 mL; BF_3_ 14% in methanol, Sigma-Aldrich Inc.,St Louis, MO, USA) and again heated for 20 min at 80 °C. After addition of saturated NaCl (5 mL) the FAME upper layer was collected and analysed using gas chromatography-flame ionisation detection (Agilent 6890N GC with an Agilent 7683 series auto sampler; Agilent Technologies Inc., Palo Alto, CA, USA). Sample (1 μL) was injected in a split mode of 20:1. Hydrogen, with a flow rate of 2.2 mL/min was used as the carrier gas, and separation was achieved using SGE BPX-70 column (50 m × 0.32 mm ID × 0.25 μm films; SGE, Melbourne, Australia). The column oven increased from 120 °C to 225 °C at a rate of 3 °C/min, then increased 10 °C/min to reach a final temperature of 245 °C, with a hold of 2 min, reaching a max temp of 270 °C (total run time 39 min). The injector and flame ionisation detector were 240 °C and 250 °C respectively. The FA were identified using the FAME reference standard (FAMQ-005, Accustandard Inc., New Haven, CT, USA). Duplicates of three crude oil samples were conducted.

### 2.4. Protein Separation and Properties

**Figure 1:**
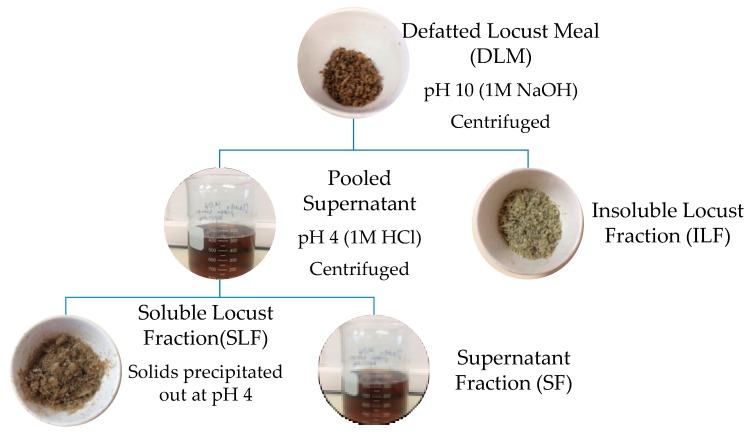
A schematic representation of the methodology used to extract *L. migratoria* protein fractions.

Protein fractions were extracted from defatted locust meal (DLM) using an adapted alkali isoelectric precipitation method outlined by Amza [27] and Tirgar et al. [28]. Figure 1 below shows a basic schematic diagram of the various fractions extracted. DLM (11 g) was mixed with deionized water (EMD Millipore Corporation, Milli-Q Advantage A10 Water System, Merck, Billerica, MA, USA) at 5% (*w*/*v*) and stirred using an overhead stirrer (IKA, RW20, Total Lab Systems, Auckland, New Zealand). The pH was adjusted to 10.0 [15] using 1 M NaOH (VWR International, LLC, Global Science Ltd.,Auckland, New Zealand) and stirred at 900 rpm for 1.5 h. The solution was centrifuged at 15,300 g for 30 min at 4 °C (Beckman JA-14 M/E, Beckman Coulter Ltd., Palo Alto, CA, USA). This was repeated once more, ultimately forming two fractions (Figure 1); solid residue referred to as the insoluble locust fraction (ILF), and the upper liquid layer (pooled supernatant). The supernatant’s pH was adjusted to 4.0 using 1 M HCl and stirred at 900 rpm for 1 h. pH 4 and 10 were chosen following similar methods conducted by Bußler et al. [15]. After centrifugation at the same conditions as above, the residual precipitated protein was re-suspended twice in acidified water (pH 4) and centrifuged at similar conditions for 30 min. This formed two fractions; precipitated locust protein referred to as soluble locust fraction (SLF) and the supernatant fraction (SF). SLF was adjusted to pH 7 using 1 M NaOH. The resulting three fractions; ILF, SLF and SF, were freeze dried and stored in a desiccator at room temperature for further analysis. Protein and fat contents were identified using the same methods as proximate analysis. Yield of each fraction is based on dry weight basis compared to total initial weight. Results are also presented as a percentage of protein recovery in which the protein content of each fraction is divided by the initial total protein content of the sample tested (after defatting).

#### 2.4.1. Colour

A Hunterlab spectrocolourimeter (MiniScan XE Plus, Reston Virginia, USA) was used to determine the colour of the LM and the three fractions (SLF, IFL and SF). Equipment was set at illuminant D65, 22 mm aperture, and 10° observer. Calibration was run using black and white tiles in accordance with the standard manual. The averages were used to calculate the change in colour (ΔE) using the following equation:

(1) $$\Delta E=\sqrt{\left({\Delta L}^{2}+{\Delta a}^{2}+{\Delta b}^{2}\right)}$$

where: L* value indicates lightness, a* value indicates red/green colour and b* value represents the yellow/blue colour of the sample.

#### 2.4.2. Water and Oil Holding Capacity (WHC & OHC)

The water and oil holding capacity was determined using adapted methods outlined by Tirgar et al. [28]. Deionised water (5 mL) or cold pressed rape seed oil (5 mL; Zeaola^TM^, New Zealand Vegetable Oil Ltd., Christchurch, New Zealand) was added to 0.25 g of LM, DLM, SLF, ILF or SF in 15 mL centrifuge tubes. The mixture was agitated at 300 rpm for 30 min in an orbital mixer incubator (Ratek, VIC, Australia), then centrifuged at 1610 g for 10 min at 4 °C (Beckman, Coulter Ltd., Pao Alto, CA USA). The supernatant volume was decanted and measured using a measuring cylinder. The water (WHC) and oil holding capacity (OHC) is expressed as the amount of water or oil (mL) that can be held per gram of sample (g) and was calculated using the following equation:

(2) $$\mathrm{WHC}/\mathrm{OHC}\;\left(\mathrm{mL}/g\right)=\frac{\left(\mathrm{volume}\;\mathrm{of}\;\mathrm{water}\;\mathrm{added}\left(\mathrm{mL}\right)-\mathrm{volume}\;\mathrm{of}\;\mathrm{water}\;\mathrm{in}\;\mathrm{the}\;\mathrm{supernatant}\;\left(\mathrm{mL}\right)\right)}{\mathrm{weight}\;\mathrm{of}\;\mathrm{sample}\;\left(g\right)}$$

### 2.5. Data Analysis

Measurements were reported as an average mean ± the standard deviation (SD). Statistical software SPSS (version 23, IBM Corporation, Armonk, NY, USA) was used to determine significant differences (*p* < 0.05), determined by one way ANOVA and Tukey’s post hoc test.

## 3. Results

### 3.1. Proximate Composition

Proximate composition (based on dry weight) is shown in Table 1. Protein was the most abundant nutrient followed by fat, carbohydrate and ash.

Insect composition varies largely due to species, diet, habitat, metamorphic stage, and processing method [2,5]. Therefore, the limited data concerning *L. migratoria* also varies. The crude protein, fat, and ash content of the present study were similar to results reported by Osimani et al. [8] for whole dried locusts (*Locusta migratoria*) sourced from The Netherlands. The crude protein content (50.79%) was also similar to reports by Mohamed [6], while other studies reported higher percentages, ranging from 55.5% to 65.9% [7,18,19].

The fat content (34.9%) was much higher than the average (13%) for orthopteran species (locust, crickets, grasshoppers) [5]. A study on adult *L. migratoria* conducted by Oonincx and van der Poel [7] did identify higher numbers (18.6% to 29.6%), with higher fat and lower protein contents acknowledged after wheat bran was added to their diet. Consequently, the grass and ground oat diet of the locusts in the current study would have impacted the protein and fat content found. Furthermore, the locusts were constantly active due to a constant artificial light source, and were domestically reared in incubated plastic boxes. This could alter the fat content identified, compared to literature that sourced insects from the wild [5].

The ash content was lower than reported contents for *L. migratoria*, ranging from 3.3% to 5.72% [6,7,8,18,19]. The mineral content varies depending on species and other factors such as habitat, diet, and season. Mohamed [6] found high amounts of phosphorous but low amounts of all other nutrients. Whereas, Kouřimská et al. [29] identified high amounts of iron (8–20 mg) in *L. migratoria*. Further research concerning the mineral content and bioavailability would provide more in depth information about locusts’ health benefits.

In general, the carbohydrate (CHO) content of orthopteran species is low. Mohamed [6] reported 4% CHO and 14% fiber in migratory locusts from Sudan. Although the present study did not separately measure the fiber content, reports indicate a large portion of this is sourced from the polysaccharide chitin, found in the exoskeleton of insects [6]. Chitin is an interesting fiber, with recent studies identifying populations that commonly consume insects and other chitinous foods, can contain the enzyme chitinase, indicating it may be digestible for certain consumers [30]. Further investigation into chitin and its digestibility and nutritional benefits are needed with no data concerning migratory locusts currently available. Overall, the high protein, fat, and fiber content found in the present study outline the nutritional benefits of adding locusts to your diet.

### 3.2. Fatty Acid Composition

When investigating nutritional benefits, locusts also contain appreciable amounts of essential fatty acids known for their health benefits. The FA composition of crude locust oil from *L. migratoria* is shown in Table 2. The chain lengths ranged from 14 to 18 carbons. To the authors knowledge, only Mohamed [31], Osimani et al. [8] and Ramos-Bueno et al. [32] have investigated *L. migratoria*. As a general trend, data was consistent with findings, with some differences in saturated fatty acid (SFA) and polyunsaturated fatty acid (PUFA) contents identified. Oleic acid is the most abundant FA documented in migratory species, followed by palmitic acid, leading to reasonably similar overall SFA and MUFA contents. α-linolenic acid (omega-3) content (15.7%) in the present study was within the range identified in literature (13.9% to 16.2%). However, Osimani et al. [8] found significantly lower content (3%) in whole dried locusts sourced from Netherlands, which consequently leads to a large variation in overall PUFA content of *L. migratoria* generally found in literature (12.5% to 32.3%). Moreover, linoleic acid (omega-6) in the present study was also within the range of 5.2% to 18.3% [8,31,32].

Many variables influence the FA composition of locusts. For example, the study conducted by Osimani et al. [8] found large differences in SFA and PUFA contents of whole dried locusts samples sourced from either Belgium or the Netherlands. The FA composition of locusts or other insect species is significantly influenced by their diet. Insects that consume grass or plants containing high PUFA are linked to high α-linolenic acid content in the FA composition [33]. Dreassi et al. [34] found yellow mealworms fed on wheat, oat, cornflour, and chickpea flour had much 10% higher PUFA’s content compared to mealworms fed on 100% oat flour.

Subsequently, the diet of locusts can be advantageously designed to optimise the omega-3 and PUFA content in order to produce a healthy lipid source. Still, locusts from the present study provided desirably low SFA/UFA and n−6/n−3 ratios of 0.58 and 0.55, respectively. The recommended n−6/n−3 ratio is around one, which is far from reach for many Western consumers who consume too much omega-6 in their diets, leading to ratios above 15/1 [35]. High omega-6 consumption in vegetable oils and grains for example, has been linked to various health diseases such as cardiovascular, autoimmune disease, cancer, and inflammatory diseases. Greater consumption of omega-3 rich lipids and food sources that have lower n−6/n−3 ratios can help reduce risk of these chronic diseases [4,35,36]. Lipid extraction is often a by-product of protein extraction making locust oil a sustainable and healthy alternative lipid and food source. In another study conducted by the researcher, consumer acceptance of locust oil was explored through focus group discussions. It found the omega-3 content, as well as the sustainability or novelty of locust oil is an attractive feature for some participants, indicating these features should be appropriately marketed and explored in order to improve acceptance.

### 3.3. Protein Properties

#### 3.3.1. Protein Content and Yield of Extracted Fractions

Yield and protein recovery of the three fractions, plus the protein content of LM and extracted fractions are displayed in Table 3. The protein contents of the three fractions were not significantly different to each other (*p* > 0.05, *p* = 0.073). After processing, their combined average protein content (73.6%) was 23.8% higher than the LM, illustrating significantly higher (*p* < 0.05, *p* = 0.000) protein content than whole locusts.

Similar to the current findings, Zhao et al. [11] and Purschke et al. [19] found protein content to 82% for yellow mealworm and migratory locust. Alternatively, research investigating other insect species found slightly lower values ranging from 65–75% for insoluble, 58–69% for soluble, and 50–61% for supernatant fractions [12,16]. Chitin and chitin-bound protein that is consequently indigestible, are likely to contribute to the protein content found in ILF [10]. Although not the focus of the current study, this can lead to lower digestibility compared to supernatant and pellet (soluble) fractions, as documented by Yi et al. [13].

The protein recovery of each fraction is expressed as a percentage compared to the initial protein content of the sample (DLM). The total protein yield (101.66%), ILF yield, and ILF protein recovery was similar to other literature investigating various insects [12,16]. However, the low SLF (9.83%) and high SF yield (56.60%) was unexpected. Purschke et al. [19] identified a protein recovery of 51.7% of migratory locust protein concentrate after solubilisation at pH 9. Other insect studies documented soluble yields ranging from 22% to 46% [15,16] and supernatant protein yields between 17% and 23% [12]. Experimental conditions in the present study could have impacted findings. The soluble proteins may not have aggregated efficiently at pH 4, causing a large proportion to remain in the supernatant fraction, leading to a higher SF yield and protein percentage. pH 4 was used to precipitate the soluble locust protein following Bußler et al. [15] methodology on five different insects. Purschke et al. [18] later identified pH 4 as the isoelectric point for enzymatic hydrolysed migratory locust protein. pH 10 was used to solubilise the proteins following the method by Tirgar et al. [28] and pH used by Bußler et al. [15]. However, Purschke et al. [19] identified the minimum solubility at pH 5 and maximum at pH 9. Therefore the pH used based on other insect species by Bußler et al. [15] may not have been the optimal pH. Furthermore, other experimental conditions such as NaOH concentration, pH, temperature, run time, and water to sample ratio are known to have an effect on protein yields, possibly affecting the results found in the present study [11,12,15,18,19].

#### 3.3.2. Colour

The colour of the various fractions can impact the consumer perceptions and acceptance when it is used in products, so is important to consider before product development. The colour of each fraction and LM are shown in Table 4. Processing of the LM did affect the appearance of the three fractions for L*, a*, b* and ∆E values (*p* < 0.05, *p* = 0.000). SF was the lightest sample, followed by ILF, SLF, and finally LM was the darkest.

To the authors knowledge no other literature has investigated *L. migratoria* protein isolate colouration. Overall, the locust fractions were darker compared to *A. domesticus* (house cricket) aqueous extracted fractions (L* 58.53–65.50) investigated by Ndiritu et al. [16]. The same study also found no difference between fractions, unlike Bußler et al. [15] that identified lighter insoluble samples, similar to current findings.

The extracted fractions were slightly more yellow compared to the LM. The brown and yellow colour could be due to lipophilic melanoidins, which during defatting and consequent protein extractions may be removed, creating higher a* and L* values [37]. This idea is consistent with other studies, that found a lighter defatted sample compared to whole and extracted fractions [15,16,38]. Colour change could also be attributed to enzymatic browning as a result of processing conditions such as extraction at certain pH values degrading proteins producing brown pigmentation [15,38]. 

Furthermore, ILF also had much lower a* value (1.67) compared to all other samples, indicating greater green colouration. Consequently, ILF also had a higher overall colour change (∆E 23.44) compared to SLF and SF, and much higher than insoluble yellow mealworm colour change (∆E 9.1) identified by Bußler et al. [15]. The green colouration could be due to a common green pigment identified in insects called insectoverdin. It is a mixture of two chromoproteins, one yellow component from carotenoids and the other a bile component (blue) creating the green colour in locusts [39]. However, pigmentation in insect changes depending on species, life stage, and habitat therefore differences in colour is expected [38].

#### 3.3.3. Water and Oil Holding Capacity

**Figure 2:**
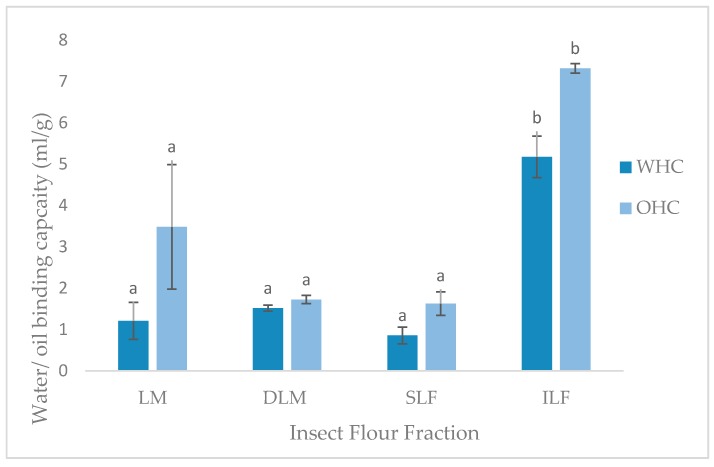
Water and oil holding capacities (ml/g) of locust meal (LM), defatted locust meal (DLM), soluble locust fraction (SLF), and insoluble locust fraction (ILF). Letters represents significant (*p* < 0.05) differences. Error bars represent SD (*n* = 2).

The water (WHC) and oil holding capacity (OHC) is expressed as the amount of water or oil (mL) that can be held per gram of sample. As shown in Figure 2, WHO and OHC values of LM, DLM and SLF were not significantly different to one another, as well as other insect species investigated [11,16]. Unexpectedly, the ILF had significantly higher (*p* < 0.05, *p* = 0.000) WHC and OHC of 5.17 mL/g and 7.31 mL/g, respectively. A study conducted by Bußler et al. [15] found whole and defatted yellow mealworm samples had higher WHC compared to the insoluble and soluble fractions, contradictory to the present study.

The ability to hold water against gravity is an important application in certain food areas. The water includes bound, hydrodynamic, capillary and physically entrapped water [15]. The ability to hold this water is dependent on many factors such as the amino acids, charges, conformation, and hydrophobicity of the protein, pH, temperature, ionic strength and protein concentration [11]. The reason for the high WHC of the insoluble locust fraction is unknown. The porous structure or large particle size, due to absence of sieving during preparation, could have caused greater entrapment of water into the particle domains. Furthermore, larger particle size has been linked to higher contents of chitin, as found in *T. molitor* grist [40], which could additionally entrap the water into the ILF matrix, creating the high WHC result found. Ability of locust fractions to hold water illustrates protein-water interactions and can be used to interpret other functional properties such as solubility, emulsification, viscosity, gelation, and foaming [11].

The high ILF OHC result is also inconsistent with the literature. Contradicting the current results, Bußler et al. [15] found fat binding capacity (g/g) was significantly higher in the soluble and lower in insoluble fractions of yellow mealworms. The current findings could again be due to the larger particle size and porous structure, causing physical entrapment of oil [11]. Additionally, higher OHC is linked to non-polar side chains in the protein structure, which was not investigated in the current study. Research on this is limited and further information on the water and oil holding capacity of protein extracts from *L. migratoria* and insects in general is needed. Nevertheless, the WHC and OHC findings indicate the possible food applications of each fraction.

In another consumer study conducted by the researcher, it asked thirty-five participants to design an ideal hypothetical solid and liquid food product containing the ILF and SLF fractions. Participants who were open to trying new or novel foods were screened prior to investigation in order to gain insights from potential end consumers. The liquid product designs (containing soluble locust fraction) designed by participants included chocolate flavoured health drinks, protein powders, an ice cream, and a coconut yoghurt product. Solid food products (containing insoluble locust fraction) included protein packed breadcrumbs, muesli bars, cereals, bread, cookies and a nut substitute. The majority of these products were designed as a healthy, convenient, and eco-friendly snack or breakfast for active consumers or children. These results indicate the exciting future of whole or extracted locust protein as a value-added ingredient.

## 4. Discussion

Locusts are beneficial in terms of their sustainable production and nutrition. Significantly lower amounts of land, water, and feed is needed to rear edible insects compared to other livestock. Additionally, locusts contain high amounts of protein, fat, and essential fatty acids which is impacted by diet, age, habitat, and processing method. Future research investigating how the composition changes at different life stages (nymph vs adult), diets, and seasons would indicate how to optimize locust composition depending on their anticipated end use. Protein extraction after defatting and subsequent processing methods, can improve the protein content, increase water and oil holding capacity for insoluble protein fractions, and decrease the dark brown coloration of whole locusts. The insoluble fractions could be used in a variety of solid products, whereas soluble and supernatant fractions are water soluble, therefore venture into the liquid food and drink market is possible. However, the low yield and replication number in the current study warrants further investigation concerning what experimental conditions or methods would be more efficient, economical and sustainable for upscale. The use of water, solvents, and energy to produce the protein fractions does impact the initial sustainable rearing advantages. Aqueous fat extraction could be researched as a “greener” method for defatting. Moreover, information concerning the solubility, emulsification, digestibility, and amino acid profile will also enable a greater understanding of the fraction’s properties and how they can be extracted and developed to be used as food ingredients. Furthermore, locust oil is a by-product of protein extraction, and with low omega-6/omega-3 and SFA/UFA ratios, it has the potential to become a sustainable and healthy alternative lipid source. With so many potential applications, locusts are an economically and environmentally beneficial venture for the future.

## Figures and Tables

**Table 1:** Proximate composition of *L. migratoria* (% based on dry weight).

Component	Mean (%) ± SD
Crude Protein	50.79 ± 0.69
Crude Fat	34.93 ± 3.37
Carbohydrate	13.46 ± 4.53
Ash	2.42 ± 0.11

**Table 2:** Fatty acid composition of *L. migratoria.*

Fatty Acid	Mean ± SD (%)
Myristic (C14:0)	2.69 ± 0.90
Palmitic (C16:0)	27.30 ± 1.53
Palmitoleic (C16:1)	1.17 ± 0.14
Stearic (C18:0)	7.23 ± 0.99
Oleic (C18:1n9c)	37.02 ± 0.67
Linoleic (C18:2n6c)	8.94 ± 0.59
α-Linolenic (C18:3n3c)	15.64 ± 2.68
Total SFA	37.22
Total MUFA	38.49 ± 1.09
Total PUFA	24.57 ± 3.27
SFA/UFA	0.58
N−6/n−3	0.55

SFA (saturated fatty acid): C14:0—Myristic acid; C16:0—palmitic acid; C18:0—stearic acid. MUFA (monounsaturated fatty acid): C16:1—plamitoleic acid; C18:1n9c—oleic acid. PUFA (polyunsaturated fatty acid): C18:2n6c—linoleic acid; C18:2n3—α-linolenic acid. UFA (unsaturated fatty acids): MUFA + PUFA.

**Table 3:** Mean (±SD) protein content (%), yield (%), and protein recovery (% of total protein) of extracted *L. migratoria* fractions (based on dry weight).

Fraction	Protein Content ± SD (%)	Yield (%)	Protein Recovery (% of Total Protein)
Locust meal	50.79 ± 0.69 ^a^		
Insoluble locust fraction	81.29 ± 4.43 ^b^	37.76	39.85
Soluble locust fraction	73.64 ± 10.64 ^b^	9.83	9.64
Supernatant fraction	69.13 ± 2.89 ^b^	56.60	52.17

Protein contents (%) with different letters within column are significantly different at *p* < 0.05 (*n* = 3). Yield and protein recovery percentages, *n* = 1. Locust meal (LM); Soluble locust fraction (SLF); Supernatant fraction (SF); Insoluble locust fraction (ILF).

**Table 4:** Colour characteristics of whole and extracted locust protein fractions.

Component	∆E	L*	a*	b*
Locust meal	-	28.14 ± 2.62 ^a^	4.89 ± 0.64 ^b^	10.06 ± 1.46 ^a^
Insoluble locust fraction	9.21 ± 3.37 ^a^	35.48 ± 0.96 ^c^	5.76 ± 0.13 ^c^	15.56 ± 0.29 ^b^
Soluble locust fraction	6.94 ± 4.53 ^b^	30.80 ± 3.08 ^b^	8.87 ± 1.51 ^d^	15.09 ± 2.50 ^b^
Supernatant fraction	23.44 ± 0.11 ^c^	50.88 ± 1.26 ^d^	1.67 ± 0.07 ^a^	14.80 ± 0.42 ^b^

Data is expressed as means (%) ± standard deviation. Means with different letters within each column are significantly different at *p* < 0.05 (*n* = 3). Change in colour (∆E); Lightness (L*); red/green colour (a*); yellow/blue colour (b*).
